# Failure analysis of primary waste heat boiler tube in ammonia plant

**DOI:** 10.1016/j.heliyon.2021.e06151

**Published:** 2021-02-02

**Authors:** Husaini Ardy, Yudhistira Perdana Putra, Adimas Dwi Anggoro, Arie Wibowo

**Affiliations:** a Material Science and Engineering Research Group, Faculty of Mechanical and Aerospace Engineering, Bandung Institute of Technology, Bandung, Indonesia; b Technical Inspection Department, PT. Pupuk Kaltim, Bontang, Indonesia; c Research Center for Nanoscience and Nanotechnology, Bandung Institute of Technology, Bandung, Indonesia

**Keywords:** Failure analysis, Flow accelerated corrosion, Short-term overheating, Thin-lip rupture, Waste heat boiler tube

## Abstract

The primary Waste Heat Boiler (WHB) in an ammonia plant experienced cap leaking and the outer tube rupture after ten months since the last repair and replacement (total retubing). The leaking cap and the outer tube materials are low carbon steel SA-204 Gr. B and SA-209 Gr. T1a. The inappropriate vertical part of the leaked cap, which is 2.4 mm shorter than the design, might trigger turbulence flow inside the cap and lead to flow-accelerated corrosion (FAC), as suggested by the appearance of wall thinning and horseshoe pattern in the inner surface. This condition is severed by improper cap material selection with low chromium content (0.01%), which is more susceptible to FAC. The local turbulence flow might erode the oxide layer at the cap bottom and accumulate the oxide deposit around the circumference weld joint and the nearest nail spacer in the tube, represented by a thick Fe_3_O_4_ deposit. The primary WHB outer tube failure might occur due to the lack of cooling from boiler water because of cap leakage combined with a thick Fe_3_O_4_ scale deposit on the nail spacer that causes significant local temperature increases on the failed tube, which resulted in yielding and thin-lip rupture.

## 1 Introduction

**Figure 1:**
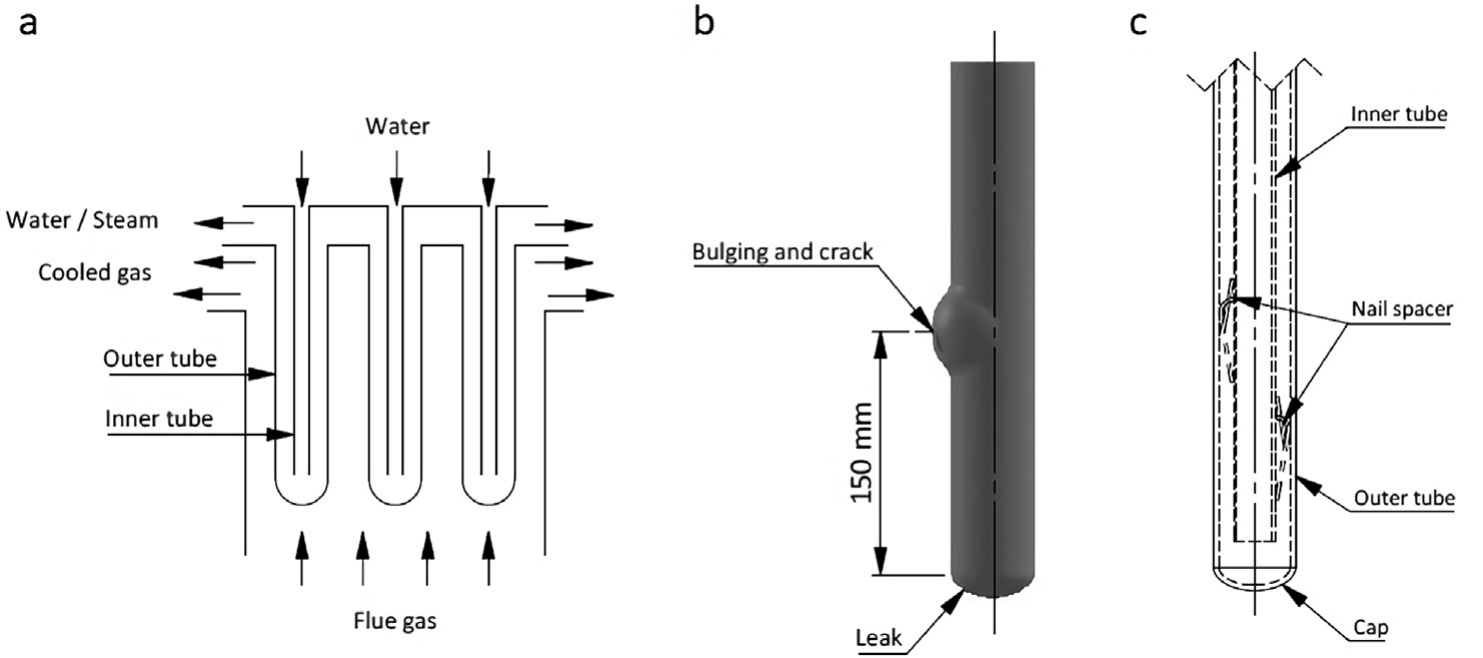
Schematic illustration of (a) primary waste heat boiler (WHB), (b) outer tubes is showing the locations of the leak, bulging, and cracking, and (c) cross-section of the tubes, showing inner and outer tubes, and nail spacer location.

Waste heat boiler (WHB) is widely used in industry because it offers an economical and efficient alternative to improve energy consumption efficiency and cost-saving by recovering heat from the exhaust gas and reuse it as much as possible to generate steam (Jouhara et al., 2018; Turner and Doty, 2007). In general, WHB is constructed from a shell with a series of tubes where the heat transfer process through the tube wall occurs between water in the tubes with flue gas. One of the essential industries that use WHB for their efficiency is the ammonia plant that generates steam using flue gas from the ammonia oxidation reactor (Abbasfard et al., 2012; Lees, 2012). The primary WHB in the ammonia plant has a vertical-bayonet type consists of inner and outer tubes, where the inner tube is in the center of the outer tube. The secondary reformer gas will be cooled in WHB before flowing to the high-temperature shift reactor, and the heat from this process will be used to generate steam (Figure 1a). Excess heat from the cooling process will produce about 10% of the total required steam for the ammonia plant.

Among all of the WHB system components, boiler tubes are critical components because failure on boiler tubes might disrupt boiler performance and even plant shutdown (Ding et al., 2017). Several failure mechanisms that commonly occurred are creeps (Liu, 2015; Ray et al., 2003), thermal fatigue (Ahmad et al., 2010; Vetriselvan et al., 2018), corrosion (Daneshvar-Fatah et al., 2013; Srikanth et al., 2003) and overheating (Dhua, 2010; Duarte et al., 2017). Because of the enormous potential for economic loss and safety risk, failure of boiler tubes in the WHB system can be minimized by understanding the potential failure root cause, particularly lesson-learned from industrial practices. A failure case from primary WHB in ammonia plant that leads to plant shutdown in January 2014 will be reported in this contribution. Failure was initiated by the leaking of a cap on the tube bottom and bulging at 150 mm from the cap (Figure 1b) in the position where one of the inner tube nail spacers was attached (Figure 1c). There are 15 nail spacers in each tube to support the inner tube and precisely maintain its position in the outer tube's center. Before plant shutdown, primary WHB had lost more than 30 tons/hour of steam, and decreasing outlet steam temperature was detected since September 2013, which might be correlated to the leakage of the outer tube cap found later during plant shutdown.

The repetitive failure occurred before tube replacement but has never been studied by the company. The company decided to study the last failure because it occurred ten months after replacement. The bottom cap leak initiated all the failure. Therefore, it is necessary to investigate the root cause of this failure to prevent similar future failures. In this research, two investigation results will be presented in sequence: the cap leakage and the outer tubes stress-rupture.

## 2 Materials and methods

### 2.1 Materials

**Figure 2:**
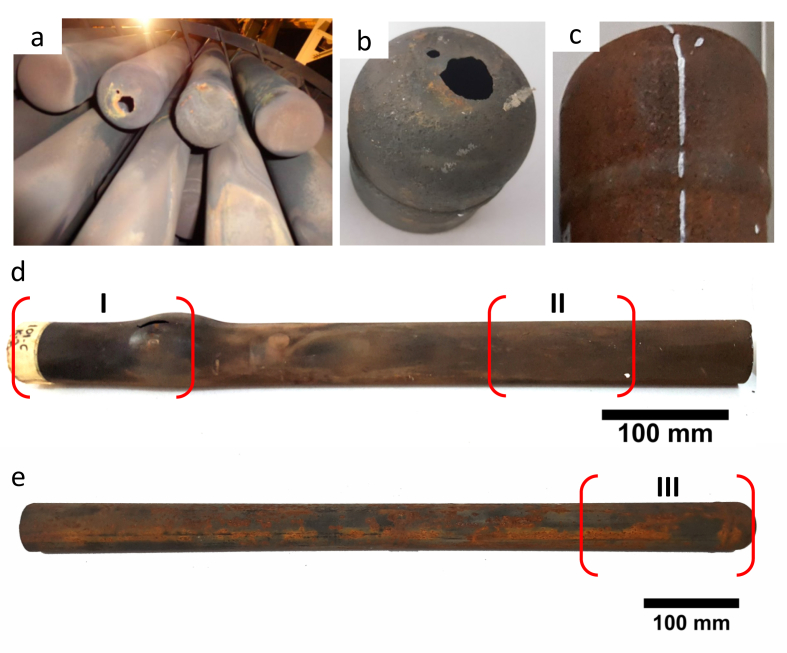
Photographs of (a) location of the leaked cap and tubes in boiler, (b) the leaked cap, (c) un-leaked cap, (d) the failed tubes at two investigation locations (sample I and II), and (e) the un-failed tubes as a reference at one location of investigation (sample III).

Figure 2a shows the locations of the failed cap and tube in the boiler. Samples for the cap's laboratory examinations were taken from leaked (Figure 2b) and un-leaked (Figure 2c) caps. While laboratory investigations of the tubes were performed at the failed outer tubes with bulging and cracking (Figure 2d) and representative of un-failed outer tubes from the same WHB bundles (Figure 2e) as the reference. Investigation of the failed tube was performed on the bulging area (sample I) and 300 mm above (sample II), while the investigation of un-failed outer tubes was only performed at the end of the tubes (sample III).

### 2.2 Operating conditions

The operating condition and specification of the outer and the inner tube materials were described in Table 1. High temperature (995 °C) and pressure (3.07 MPa) flue gas from secondary reformer was cooled by boiler water in the outer tube of WHB so that flue gas temperature at the outlet side was decreased to 525 °C. Boiler water quality was periodically inspected based on the acceptance criteria from Licensor that is shown in Table 2.

**Table 1:** Operating conditions of the primary WHB.

	Shell Side	Tube Side
Type of fluid	Sec. Reformer Effluent Gas	Boiler Water (BW)
Design Pressure, MPa	3.6	11.8
Operating Pressure, MPa	3.07	10.55
Operating Temperature, °C	995 (inlet)/525 (outlet)	314
Tube Material	SA-179 (inner tube) SA-209 Gr T1a (outer tube)	
Cap Material	SA-204 Gr B	
Tube Size, mm	25.4 OD x 1.65 thickness, 8235–8845 Length (inner tube) 50.8 OD x 3.4 thickness, 7027–7637 Length (outer tube)	
Number of Tubes, ea	421

**Table 2:** Boiler water acceptance criteria.

Parameter	Acceptance criteria
pH	8–10.2
Conductivity (μS/cm)	15–80
SiO_2_ (ppm)	0–1
PO_4_ (ppm)	3–10
N_2_H_4_ (ppm)	0.01–0.03

### 2.3 Material characterizations

The root cause of failure was explored by laboratory examinations, including visual inspection, chemical analysis, metallographic examination, hardness testing, and deposit analysis. Visual inspection was carried out to observe the outer and inner cap and tube surfaces' overall condition and differences. Visualization of the outer and inner cap and tube surfaces and the corrosion profile on the cap and tube's inner surface was carried out using a digital camera and stereomicroscope (SMZ18, Nikon, Japan). Chemical compositions of material were examined using Optical Emission Spectrometry (OES) analysis (ThermoFisher ARL 3460, USA).

An optical microscope (Eclipse MA200, Nikon, Japan) was used for metallographic examination in samples' cross-sections. The metallographic samples were prepared following ASTM E3-11 and ASTM E407-07 standards. The samples were mounted in resin, then ground and polished with sandpaper and alumina paste, respectively (ASTM, 2017a). The polished samples were etched with a 2% technical grade nital etchant solution (containing nitric acid in ethanol) (ASTM, 2015), purchased from Bratachem, Bandung, Indonesia.

The microhardness tests of cap and tube samples were conducted using Vickers Hardness Testing Machines (ZwickRoell, Germany), using 200 g of load for 10–15 s following ASTM E384-17 standard (ASTM, 2017b). The measurements in one area were performed in three different locations to obtain valid data. Elemental compositions of corrosion scales from the inner surface of the failed cap and tube were examined by Energy Dispersive Spectroscopy (EDS) analysis (Scanning Electron Microscope SU3500, Hitachi, Japan), and the compound types were examined by X-Ray Diffraction (XRD) analysis (PW3040/x0 X'Pert PRO, PANalytical, Netherlands).

## 3 Results and discussion

### 3.1 Failure analysis of cap

#### 3.1.1 Cap material

The chemical composition of the leak and un-leak caps has been examined to identify the cap material. Table 3 shows the chemical composition of both caps and their comparison to ASME SA204 grade B specification. Caps materials comply with SA204 Gr. B (ASME, 2010a) specification; however, these two caps might be coming from the different lots because the un-leak cap material is SA213 Gr. T12 (ASME, 2010a).

**Table 3:** Chemical composition of caps (weight %).

Elem Ent	Un-leak cap	Leak Cap	SA 204 gr. B Specs.	SA 213 gr. T12 Specs.
Carbon	0.19	0.19	≤0.20	0.05–0.15
Manganese	0.67	0.90	≤0.90	0.30–0.61
Phosphorus	0.022	0.017	≤0.025	≤0.025
Sulfur	0.005	0.006	≤0.025	≤0.025
Silicon	0.56	0.26	0.13–0.45	≤0.50
Molybdenum	0.45	0.54	0.41–0.64	0.44–0.65
Chromium	1.04	0.01	---	0.80–1.25

#### 3.1.2 Visual observations

As shown in Figure 2a, the cap is part of the BW tube and was welded to the tube's bottom end. Two leaks point near the centerline were found on the leak cap's bottom (Figure 2b). The morphology of the leak point suggested that the wall thinning started from the bottom inner surface.

**Figure 3:**
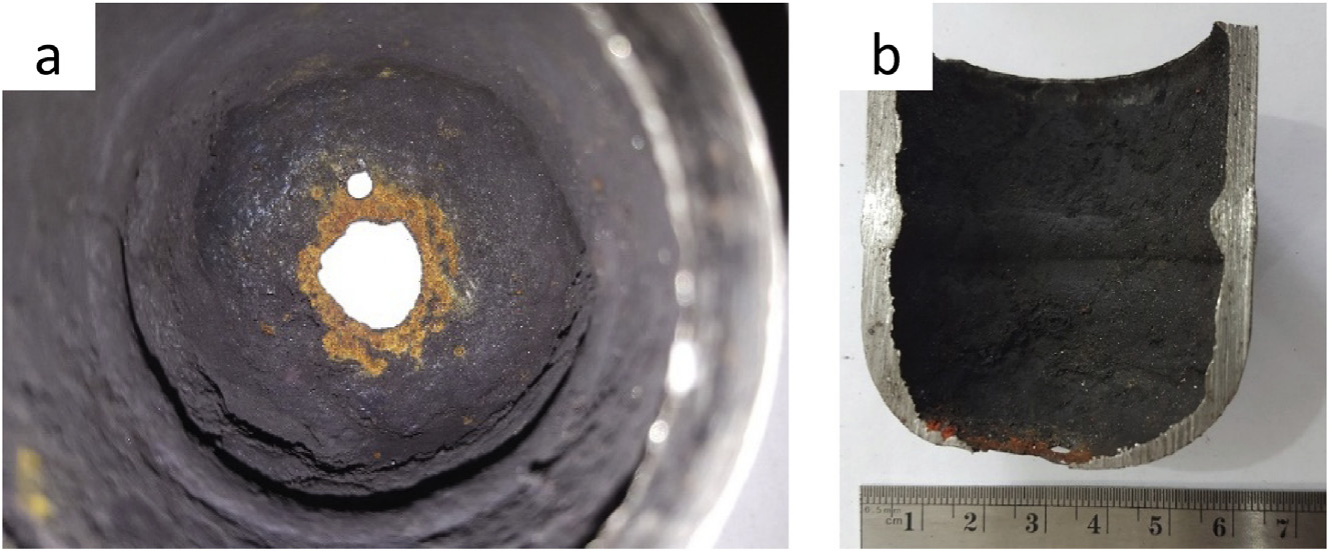
The leaking cap's inner surface view: (a) from the top side and (b) from the longitudinal section.

Since the thinning started from the inner surface, the leaking cap was sliced into two parts to observe the inner surface condition, as shown in Figure 3. The inner surface was coated by a thick deposit (Figure 3a) that reduce the cap's inner diameter. The bottom wall thinning can be seen clearly, including two leak locations at the cap's centerline. Thinning is on the bottom and symmetrical thinning on the vertical wall (Figure 3b).

**Figure 4:**
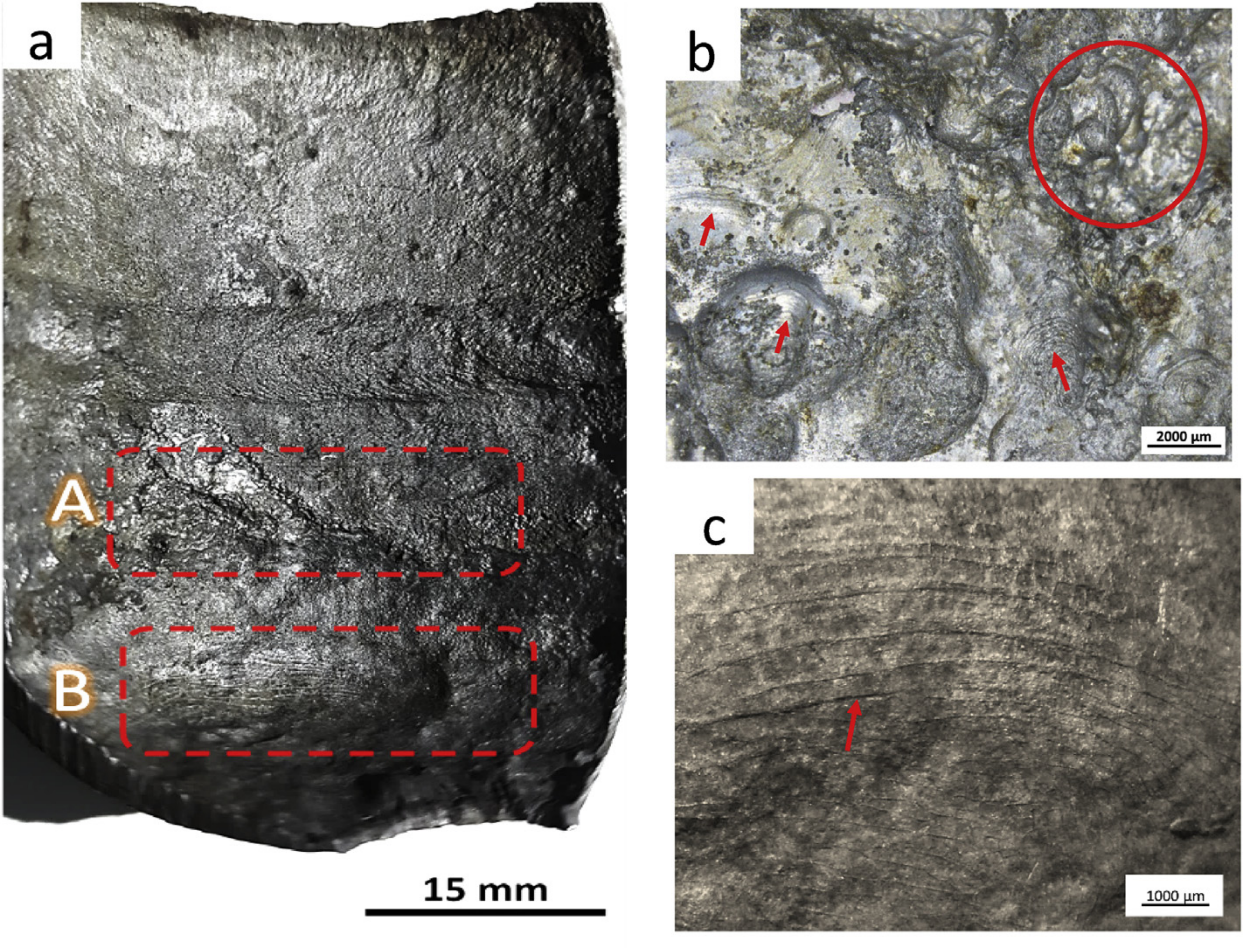
(a) Stereographs of failed cap's inner surface and higher magnification on the observation locations: (b) area A and (c) area B.

**Figure 5:**
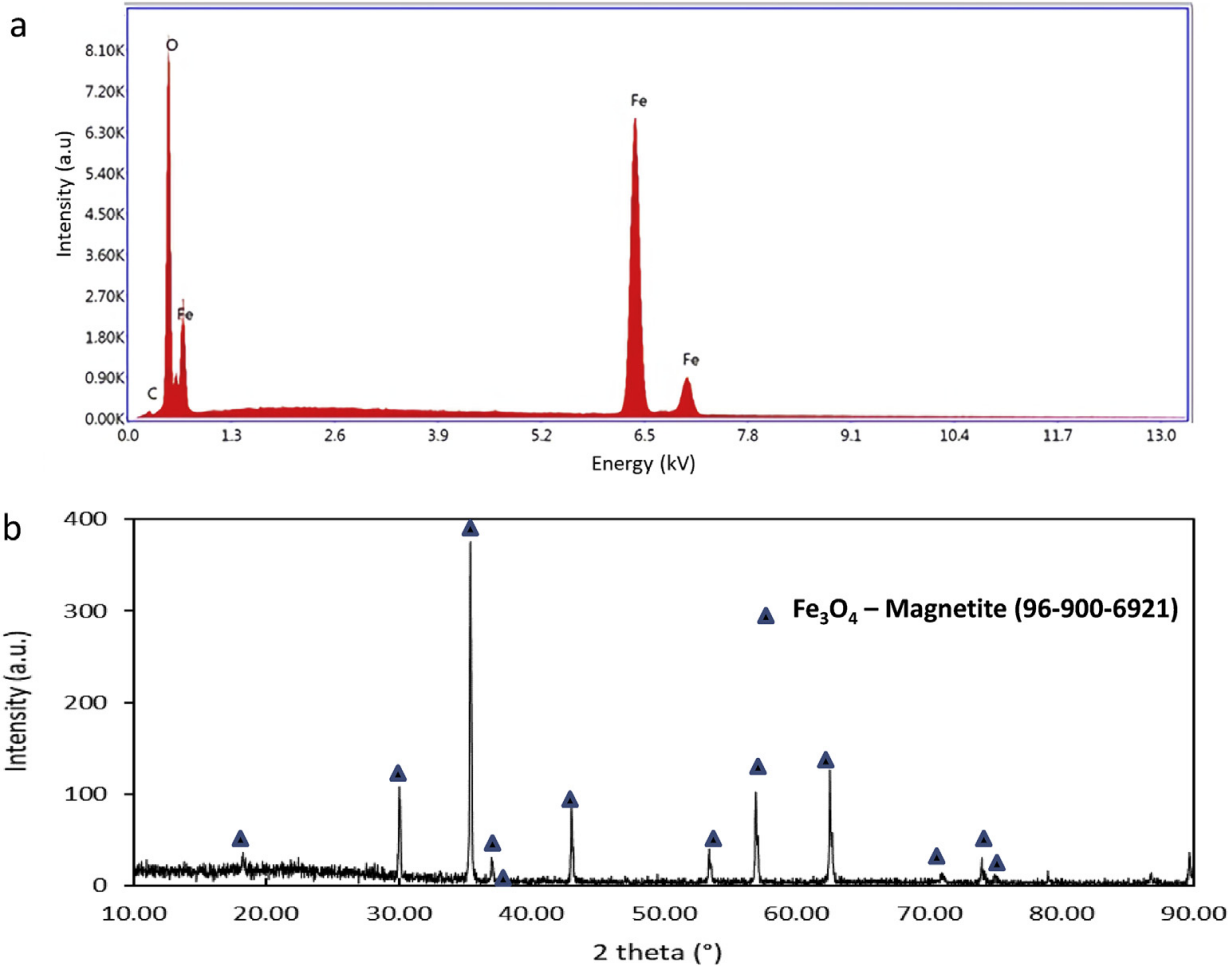
(a) EDS spectrum and (b) XRD pattern of inner cap deposit.

The root cause of thinning will be explored by observation of the leak cap's inner surface. Before the examination, the cap's inner surface was cleaned by 9% chloric acid pickling solution followed by immersion in ethanol, rinsed in flowing tap water, a final rinse with ethanol, and dried. Figure 4 shows the stereographs of the cleaned inner surface of the leaking cap. The surface profile of area A (Figure 4a) reveals the traces of erosion as shown by the presence of the horseshoe (red circle) and wavy pattern (red arrows). The other wavy profile was also observed in area B, as shown in Figure 4c. Since the horseshoe pattern (Herro and Port, 1993), or widely known as scallops (Abe et al., 2017), is one of the flow accelerated corrosion (FAC) failure characteristics, it raises the speculation that FAC might happen in the leaked cap. These typical morphologies were also observed in deaerator carbon steel pipe elbow of the fossil power plant (Abe et al., 2017), feedwater line of the atomic power station (Kain et al., 2011), and economizer inlet tube (Dooley and Lister, 2018). The wavy pattern in Figure 5c represents the existence of turbulence flow that eroded the oxide layer and thinning of the bottom cap wall.

#### 3.1.3 Deposit analysis

EDS and XRD methods examined the corrosion deposit accumulated in the weld joint area of the leaked cap's inner part to determine the corrosion species' contribution to the leak (Figure 5). Based on the EDS result (Figure 5a), the deposit element is iron and oxygen. Further examination of the deposit using the XRD method (Figure 5b) revealed that the deposited compound is magnetite (Fe_3_O_4_), as the corrosion process's product might be carried away from the bottom surface and accumulated in the weld joint.

#### 3.1.4 Shape and dimensions of cap

**Figure 6:**
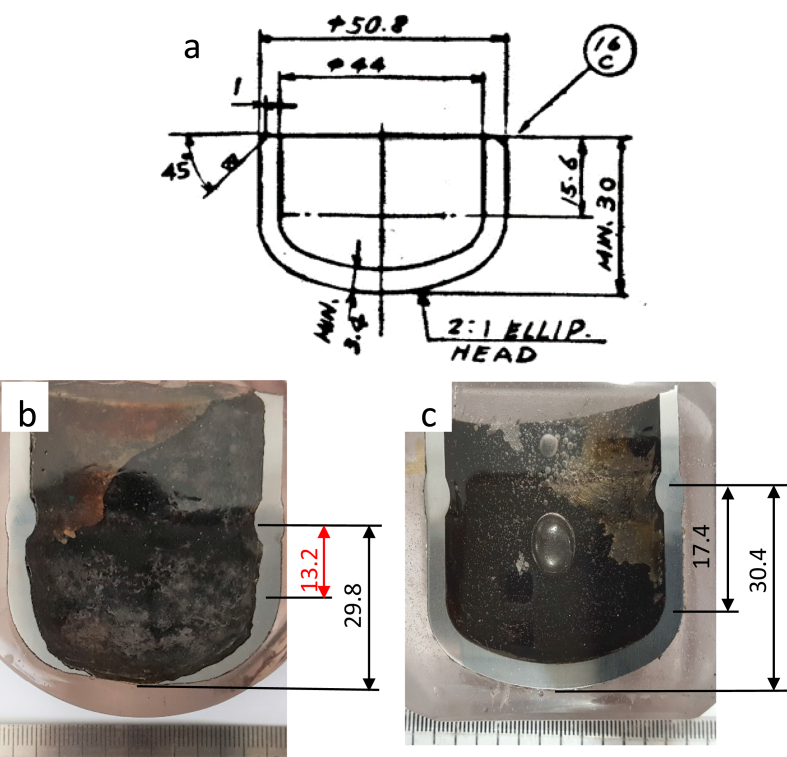
The shape and dimensions of the cap; (a) the cap's design specification, (b) leak cap; and (c) un-leak cap.

The leak and un-leak cap's (Figure 6c) internal shape and dimensions have been compared to the cap's design specification. The cap consists of the ellipse head and the vertical part, which dimensions have been specified by design (Figure 6a). The leak and the un-leak cap's dimensions fulfilled the design requirement, but the vertical part of the failed cap was 2.4 mm shorter than the design (Figure 6b), while the un-failed cap was 1.8 mm longer than the design (Figure 6b). The deviation of dimension from the design requirement might contribute to the turbulence flow and flow-assisted corrosion (FAC). Thus, the leaking cap with a shorter vertical part might suffer higher turbulence induces to failure than the un-leak cap with a longer vertical part.

#### 3.1.5 Failure mechanism of cap

Based on the above examination results, the cap's failure might be triggered by the inappropriate vertical part of the leaking cap, which is 2.4 mm shorter than design, triggering turbulence flow inside the cap and leading to FAC. The local turbulence flow will erode the oxide layer and expose a fresh surface to water and form a new oxide layer. The oxide layer's erosion and formation occurred repetitively, accumulating the oxide deposit around the weld joint and some of them progressing to the tube side, which is confirmed by the XRD result of the deposit (Figure 5b). The presence of solid oxide particles in water inside the cap will accelerate erosion until a small leak was formed in which oxygen from the air may leak into the cap and enhanced oxide layer formation (Kain, 2014). The leak size increased with erosion time and progressively increased the tube temperature because the cooling effect was diminished.

Cap material might also be crucial to their susceptibility against FAC. As shown in Table 3, both leak and un-leak caps are low carbon steel but with different chromium content. Chromium content in the leaking cap (less than 0.3%) is lower than the un-leak cap (1.04%). Consequently, the leaking cap is more susceptible to FAC than the un-leak cap.

### 3.2 Failure analysis of tube

#### 3.2.1 Visual observations

**Figure 7:**
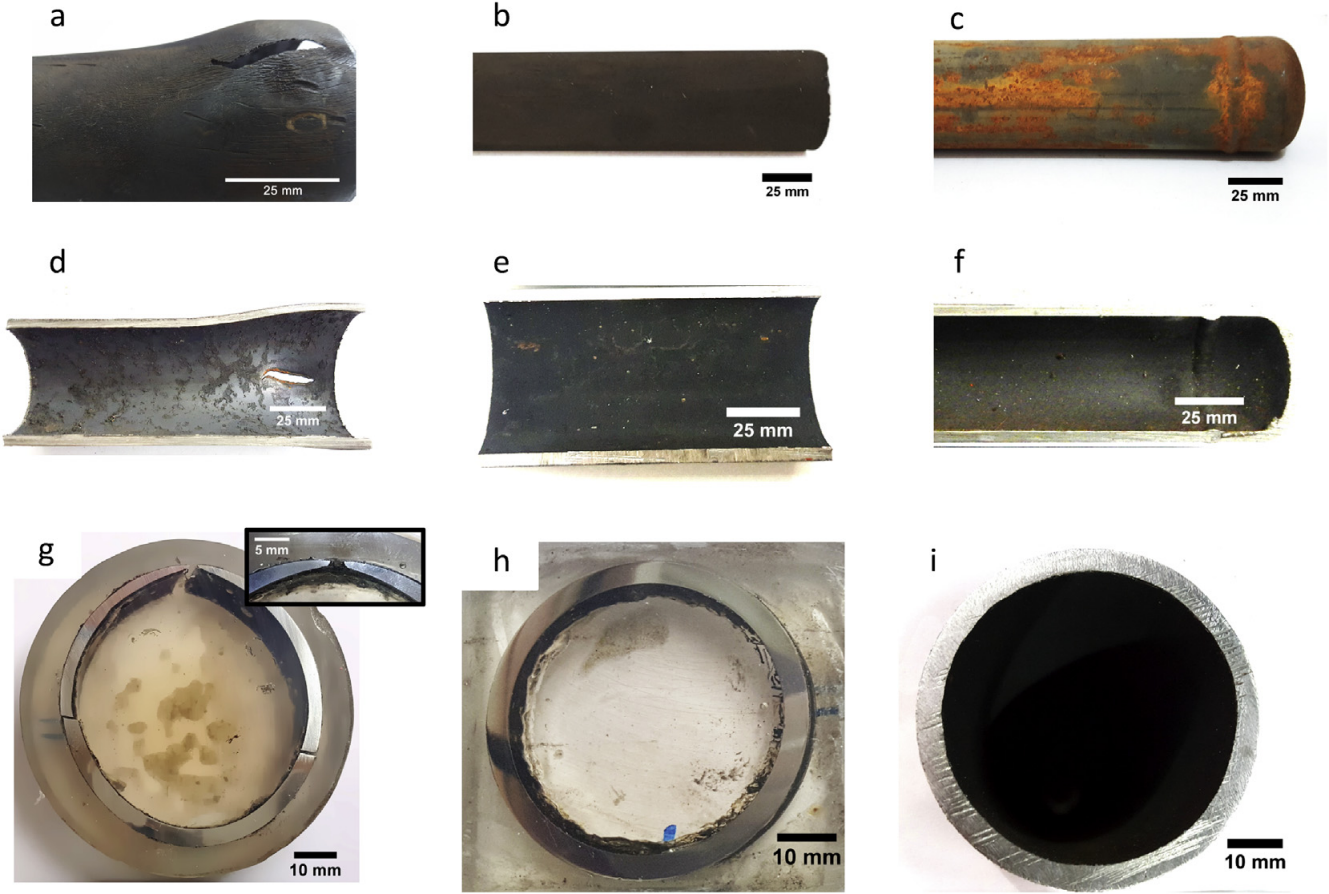
Visual observation of the outer surface of (a) sample I, (b) sample II, (c) sample III; the inner surface of (d) sample I, (e) sample II, (f) sample III; and cross-section of (g) sample I, (h) sample II, (i) sample III. Inset of image g is the edge of the bulging area that was showing thin-lip rupture.

Visual observations have been conducted to observe the outer and inner surfaces, overall condition, and tube samples' cross-section. The outer surface of all the samples is relatively clean (Figure 7a-c). A single longitudinal crack about 25 mm long and several stretch marks were found on the outer side of sample I (Figure 7a), while sample II (Figure 7b) and sample III (Figure 7c) are relatively smooth. The presence of a single longitudinal crack and several stretch marks around the crack, suggesting that crack might occur due to hoop stress (Makhlouf and Aliofkhazraei, 2015; Munda et al., 2018).

The inner side of sample II (Figure 7e) and III (Figure 7f) are smooth and clean, but scales were observed on the inner side of sample I (Figure 7d) at nail spacer position and close enough (150 mm) to the cap leak location. There is no deposit observed on the other nail spacers. Typical appearance of cross-section tube was perceived in sample II (Figure 7h) and III (Figure 7i), while sample I diameter increased due to bulging, which results in thin-lip rupture (Figure 7g and its inset).

Two typical failure mechanisms might be observed at high temperature: short term overheating and long-term rupture (creep). The short-term overheating occurred when the local tube temperature at the instant of yielding is much higher than the design, and its occurrence might appear within several minutes. While the long-term rupture usually takes a much longer time in the order of ten to twenty years because the temperature at the instant of rupture is slightly above the design (French, 1993). Short-term overheating can be distinguished from long term rupture by its appearance as the initial identification. Based on visual observation, creep characteristics, such as the absence of plastic deformation, creep voids along grain boundaries, and intergranular crack (French, 1993), was not observed in the waste heat boiler tube. In contrast, the thin-lip rupture was observed in the tube as one of the short-term overheating failure characteristics. Therefore, it raised the initial hypothesis that the tube's failure might occur due to the short-term overheating mechanism.

Further examinations for identifying supporting evidence of short-term overheating were performed to confirm this hypothesis. Short-term overheating failure can be recognized by some characteristics such as (i) the presence of knife-edge wall thinning in the crack area, (ii) excessive bulging, (iii) the formation of spheroidized carbide, (iv) high hardness in the edge of the crack and (v) thick scale on the inner surface (Hosseini and Yareiee, 2019; Munda et al., 2018; Perdomo and Spry, 2005; Prabu et al., 2019; Purbolaksono et al., 2010).

**Figure 8:**
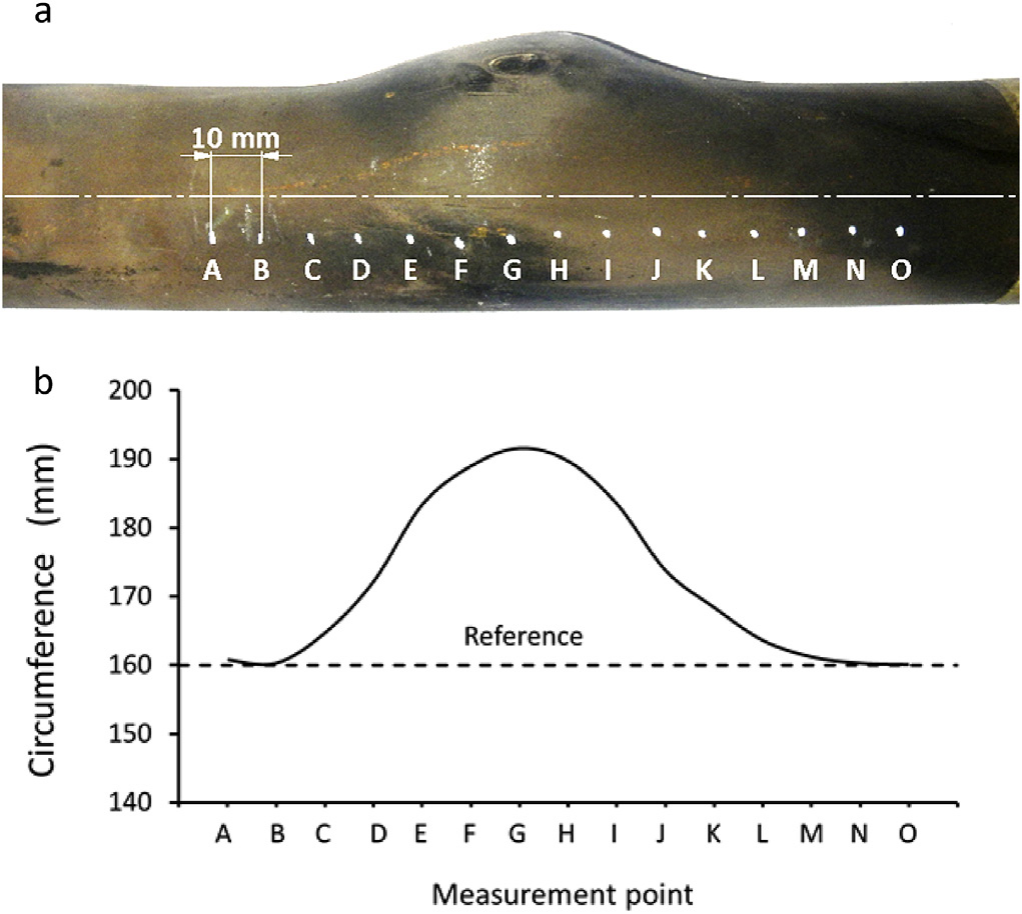
Circumference measurements of the failed tubes around the bulging area: (a) visual observation and (b) circumference measurements result of the failed tube.

Circumference measurement in several locations in the vicinity of the bulging area is performed by marking several spots, as shown in Figure 8a, and their measurement results were depicted in Figure 8b. The tube circumference specification is based on ASME Sect. II A is around 160 mm (ASME, 2010a); however, a much larger circumference was detected on the crack area (191.6 mm), which is about 19.8% higher than ASME technical specification (160 mm). This result indicated that the failed tube experienced excessive plastic deformation (yielding and bulging) before bursting. This failure can be categorized as ductile fracture, one of the typical failure characteristics due to short-term overheating because material yield strength decreased significantly (Munda et al., 2018; Purbolaksono et al., 2010).

#### 3.2.2 Chemical composition of materials

Chemical analysis results of un-failed and failed tubes and their comparison to standard specification are presented in Table 4. Chemical compositions of the tubes are complying with ASME SA-209 Gr T1a specification (ASME, 2010a). The tube material is low alloy carbon steel with low carbon content.

**Table 4:** Chemical composition (wt.%) of the tube materials.

Element	Un-failed Tube	Failed Tube	SA-209 Gr T1a Specification (ASME, 2010a)
C	0.20	0.19	0.15–0.25
Mn	0.67	0.40	0.30–0.80
P	0.019	0.020	0.025 (max.)
S	0.006	0.016	0.025 (max.)
Si	0.20	0.021	0.10–0.50
Mo	0.47	0.47	0.44–0.65

#### 3.2.3 Metallographic examination

**Figure 9:**
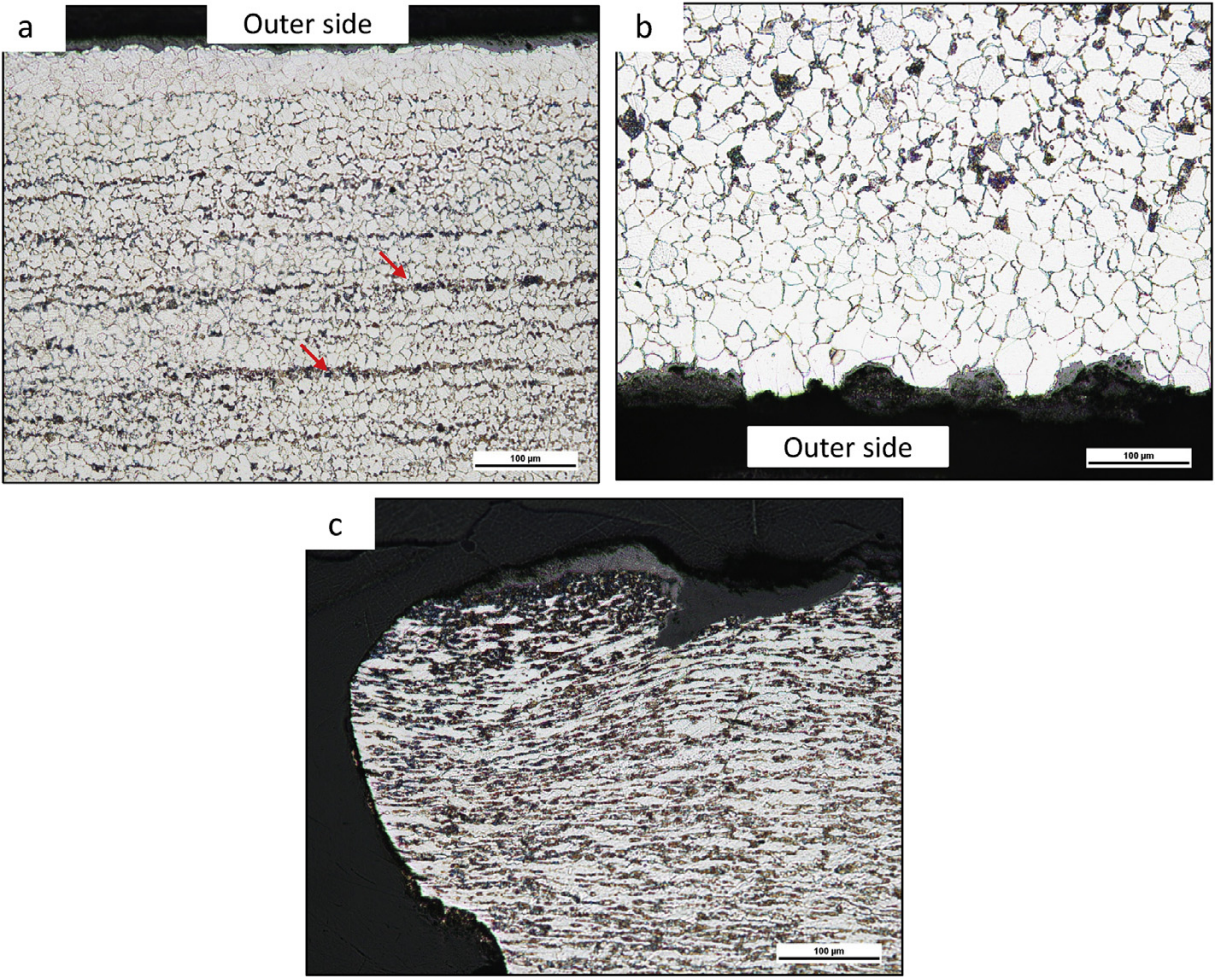
The microstructure of (a) sample III (the un-failed tube), (b) sample II (300 mm above the bulging site), and (c) sample I (at the crack tip). The red arrow in a indicates banding structures.

The microstructure analysis is crucial in identifying the root cause of high-temperature failure because some specific features such as spheroidized carbides, voids, and microcracks can be observed. The metallographic examinations in a cross-section of tube samples were performed in three locations. Microstructures of un-failed tubes (sample III) consisted of ferrite (bright) and pearlite (dark) phases with banding structures, as shown in Figure 9a. Banding structures (red arrow in Figure 9a) are typical layer structures of materials manufactured by hot rolling or hot extrusion process (Feng et al., 2015; Shih et al., 2002; Zhang et al., 2014). It is reasonable that banding structures will be present in the SA-209 Gr T1a tube because hot rolling or hot extrusion is one of the manufacturing steps in producing a seamless tube.

Microstructures of sample II (300 mm above bulging area; Figure 9b) also consists of ferrite (bright) and pearlite (dark) phases but without banding structure. Also, sample II's ferrite grain size is more significant than sample III (un-failed tube). The disappearance of banding structure and increasing ferrite grain size in sample II indicated that the failed tube was exposed to a higher temperature than the un-failed tube (sample III), even though sample II location is quite far from crack location (Verlinden et al., 2007). Furthermore, the decarburization zone in the outer surface of sample II (100 μm) is thicker than sample III (50 μm), suggesting that the failed tube suffers a higher temperature than the un-failed one. The decarburization rate increases with increasing temperature because carbon diffusion out of the surface is accelerated at a higher temperature (Liu, 2015).

Microstructures of the crack tip (sample I; Figure 9c) showed an elongated grain structure indicating high-temperature plastic deformation during yielding below the recrystallization temperature range (Verlinden et al., 2007). The presence of spheroidized carbides in the microstructure implied that the tube material had been exposed to a 440–760 °C temperature range (API, 2011). The partial spheroidization suggested that the tube material was exposed to a high temperature for some time before bulging. The tube temperature at the bulging location was determined by cap leak size and deposit thickness; therefore, there is some time lag for carbide spheroidization before reaching the yielding temperature. Once the yielding temperature was reached, the bulging occurred very rapidly, as shown by deformed pearlite in the microstructure. The local tube temperature must be lower than the eutectoid temperature (723 °C) because no Widmanstatten structure was observed. If the low carbon steel tube were exposed to the austenite region, the Widmanstatten structure would form during fast cooling from austenite in the event of bursting. Failure was not due to creep because the microstructure did not contain voids and intergranular cracks.

#### 3.2.4 Hoop stress – temperature analysis

Based on visual observation around the failed tube's crack location (Figure 7a), it is suspected that the longitudinal crack might occur due to hoop stress. The magnitude of hoop stress on the outer tube ($\sigma_{H}$) during operation can be calculated using Eq. (1) as follow (Ibrahim et al., 2015):

(Eq. 1) $$\sigma_{H}=\frac{\left(P_{i}-P_{o}\right)d}{2t}$$

Where $P_{o}$ is external pressure (shell side), $P_{i}$ is the internal pressure (tube side), d is the inner diameter, and t is wall thickness. Based on the operating condition parameters presented in Table 1, the outer tube's hoop stress was 48.4 MPa.

Considering that ductile fracture, an excessive plastic deformation before bursting that occurred because material yield strength decreases at high temperature, was observed in the failed tube (Figure 8a), a comparison between yield strength and the calculated hoop stress was performed. Generally, bulging should not appear at boiler water temperature (314 °C) because the yield strength of the material at 314 °C (181 MPa; (ASME, 2010b)) is much higher than the calculated hoop stress (48.4 MPa). However, bulging appeared in the failed tube, and spheroidization (Figure 9c) was observed in the crack tip, indicating that the actual local temperature might be much higher than the operation temperature (314 °C). Unfortunately, the actual local temperature can not be predicted because the yield strength data is only available up to 525 °C. Since yield strength of the tube at 525 °C (143 MPa; (ASME, 2010b)) is still higher than the calculated hoop stress (48.4 MPa), and the microstructure observation (Figure 8) revealed that the eutectoid temperature (723 °C) was not reached, it could be estimated that the actual local temperature of the tubes might be in between 525 and 723 °C. This estimated temperature is significantly higher than the design (314 °C), thus supporting the initial hypothesis that the failure occurred due to short term overheating mechanism.

#### 3.2.5 Hardness properties

Another characteristic of failure due to short-term overheating is increasing hardness near the crack area because of martensite formation during bursting. The current tube material is low carbon steel; therefore, hardening by martensite formation will not occur, but hardening due to plastic deformation is possible when the deformation occurred below the recrystallization temperature. Microhardness tests were performed on the near crack tip of sample I and the inner and outer part of samples II and III. A summary of the average sample hardness at various locations is presented in Table 5.

**Table 5:** The average hardness of tube materials.

Samples	Location Point	Average hardness (VHN)
Sample III (un-failed tube)	Inner surface	153 ± 2
	Outer surface	140 ± 3
Sample II (300 mm above bulging area)	Inner surface	141 ± 3
	Outer surface	120 ± 3
Sample I (bulging area)	Near crack tip (left)	171 ± 4
	Near crack tip (right)	164 ± 3

The average hardness at the inner surface of sample III (the un-failed tube; 153 ± 2 VHN) is relatively close to the standard hardness of SA-209 Gr T1a for thickness less than 5.1 mm (156 VHN (ASME, 2010a)). The average hardness at the inner surface of sample II (300 mm above bulging area; 141 ± 3 VHN) is lower than sample III, suggesting that the failed tube has been exposed to higher temperatures that lead to material softening (Prabu et al., 2019). These data were consistent with sample II's microstructure (Figure 9b), revealing a larger grain size and spheroidized carbides. On the other hand, sample II has been exposed to a higher temperature than sample III (Callister and Rethwisch, 2015).

The average outer surfaces hardness of sample III and II (140 ± 3 and 120 ± 3 VHN, respectively) are lower than their inner surface because the outer surface has been exposed to high-temperature flue gas, as indicated by the presence of a decarburization zone in the microstructure (Figure 9a and b respectively). The average hardnesses of both crack tips in sample I (164 ± 3 and 171 ± 4 VHN) were above the standard hardness requirement due to strain hardening after localized plastic deformation during bursting (Purbolaksono et al., 2010). This result is the additional evidence that was supporting the hypothesis about short-term overheating as the root cause of bulging and cracking in this case.

#### 3.2.6 Scale analysis and their origin

**Figure 10:**
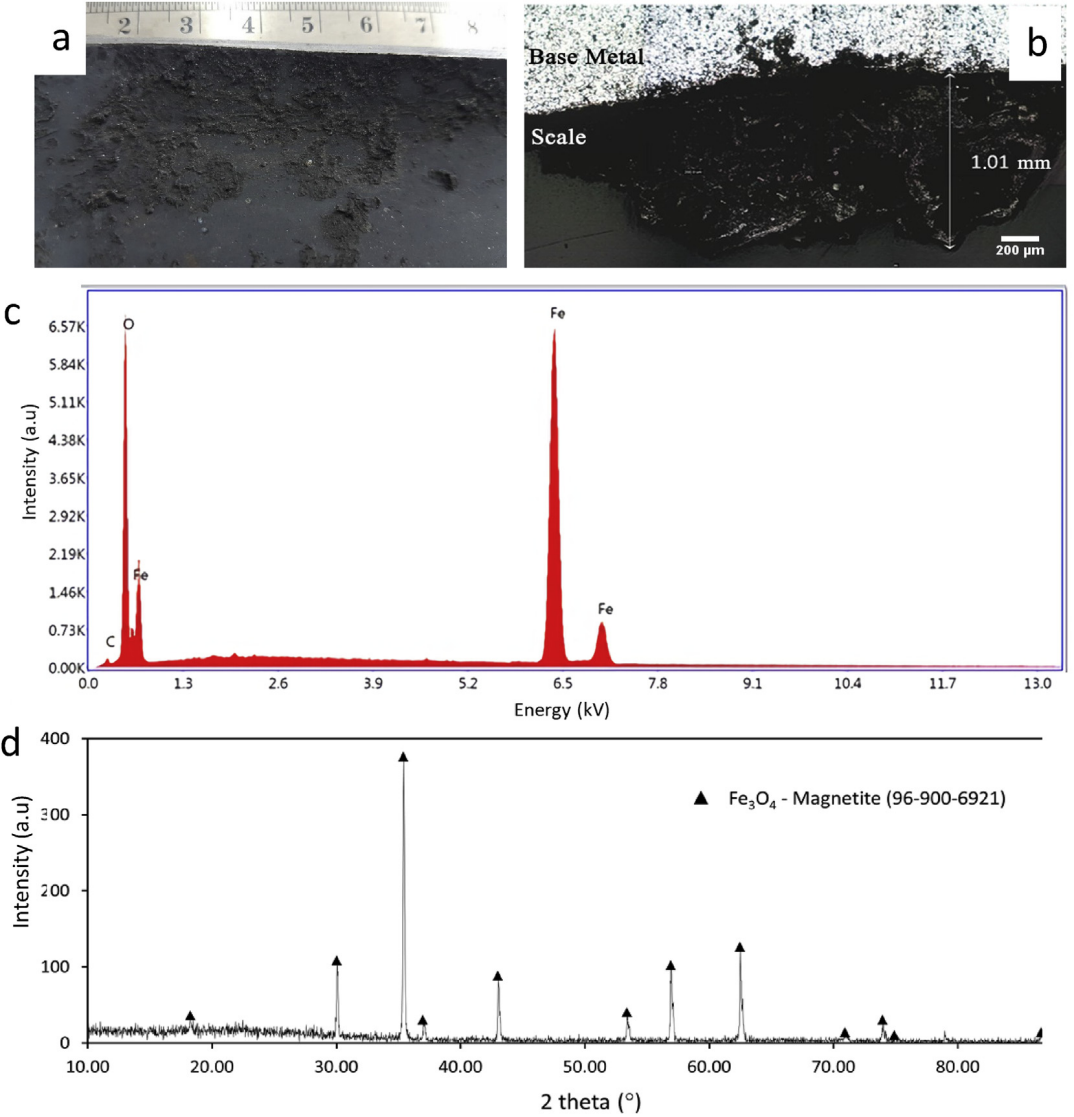
(a) Visual observation of inner side of the failed tube across of crack site, (b) optical microscope observation of a cross-section of the inner failed tube containing scale, (c) EDS results of the scale, and (d) XRD pattern of scale.

As shown in Figure 7d, scales were observed on the bulging area's inner surface (sample I). Massive and thicker scales were found around the cracked area in sample I (Figure 10a), where the nail spacer is located. The sample's microstructure cross-section unveiled thickness of the scales was about 1.01 mm (Figure 10b). The presence of a thick scale on the bulging area's inner surface is the other evidence that promotes short-term overheated phenomena.

Elemental analysis of the scales using the EDS method (Figure 10c) revealed that the dominant element in the scales is iron (73.6%) and oxygen (23.3%). No other common scale-forming elements such as phosphorus (P) or silica (Si) were found when boiler water was poorly treated, which means that water treatment for boiler water is appropriate enough to remove P and Si. Further characterization using the XRD method unveiled that only magnetite (Fe_3_O_4_; 96-900-6921) was found in the scales (Figure 10d).

**Figure 11:**
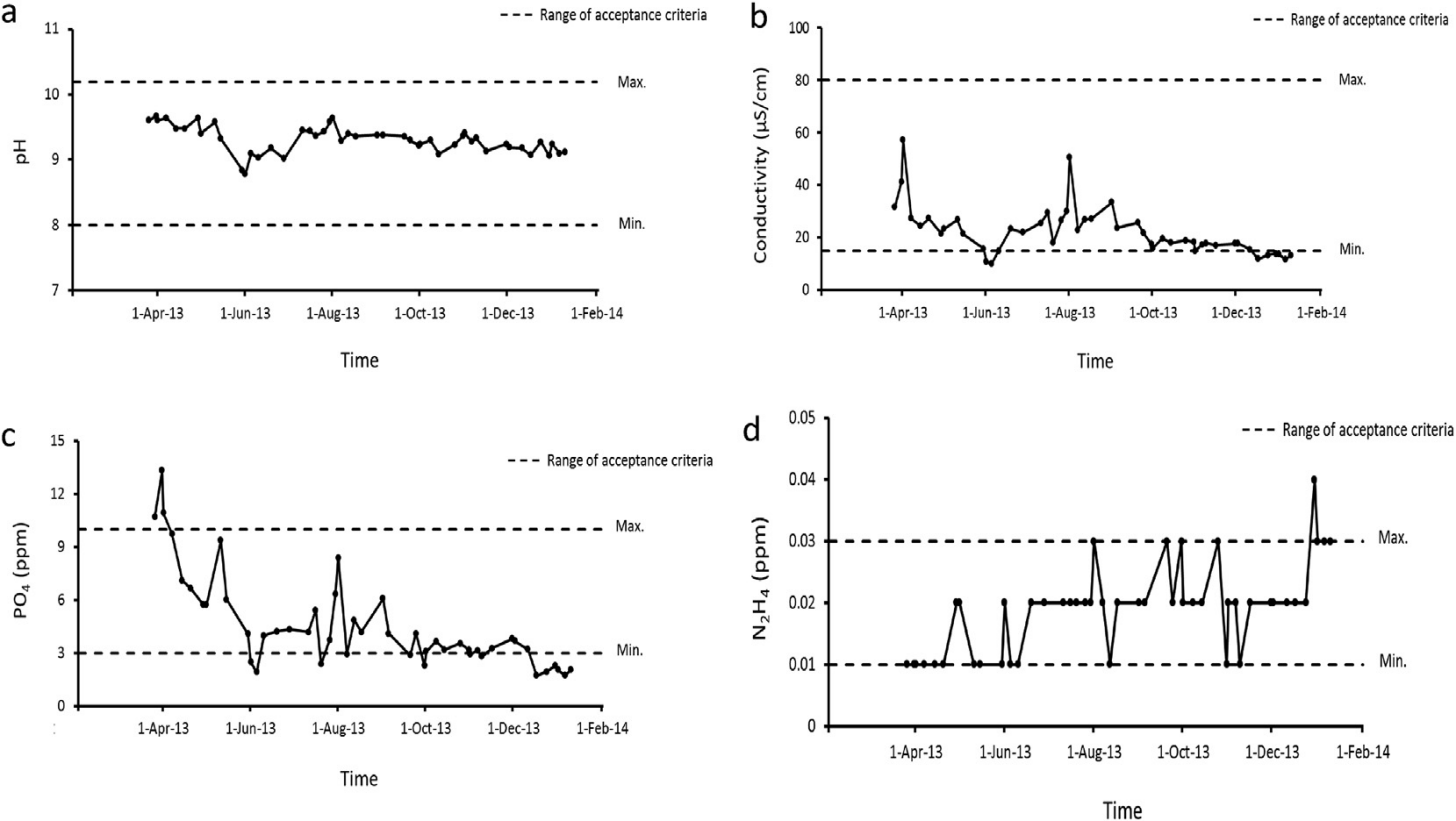
Water quality parameters from April 2013 to February 2014: (a) pH, (b) conductivity, (c) phosphate, and (d) hydrazine.

The presence of scales reduces heat exchange process efficiency significantly due to their low thermal conductivity. Therefore, an evaluation of boiler water quality, piping, and boiler system equipment to find a potential scale formation source was performed. Based on the water quality evaluation from April 2013 to February 2014 (Figure 11), all the parameters (pH, conductivity, phosphate, and hydrazine) were still in the acceptable criteria range. Thus, the scale formation problem might not be originated from low water quality. There are two possible sources of Fe_3_O_4_ deposits, from a deaerator shell which previously damaged due to erosion-corrosion and from backflow of deposit from the cap bottom before cap leakage. The corrosion product from the deaerator shell might be carried away, circulated to the boiler system, and end up in the primary WHB. The nozzle steam drum to the Primary WHB was at the lowest position in the steam drum to allow sediment to be carried to the primary WHB. However, failure was not found on the other nail spacers along the tube. Backflow of deposit from the cap bottom before leakage is the possible source of deposit scale because failure location is only 150 mm above the cap bottom. The nail spacer at the failure location trapped and accumulated this deposit scale.

### 3.3 Failure mechanisms of the tube

The laboratory test results show that the tube failure might occur due to a substantial local temperature increase. The failure was started by the cap bottom leakage and deposition of eroded Fe_3_O_4_ particles from the cap bottom backflow, trapped and accumulated on a nail spacer located 150 mm above the cap bottom. Cap leakage induces a substantial decrease in water flow combined with a thick scale layer that significantly reduced heat transfer between the flue gas and boiler water and exposed the tube wall to high temperature, leading to instant bulging and rupture.

## 4 Conclusions and recommendations

Failure of the primary WHB outer tube might be initiated by the faulty cap geometry and improper cap material that induce FAC and leakages at the cap bottom. The eroded surface deposit was accumulated, and some of them were progress to the tube and accumulated at the closest nail spacer. Deposit accumulation along the circumferential weld joint decreased the inner diameter and affected the flow profile, enhancing turbulence flow on the cap bottom. The leakage of cap bottom, which reduced the boiler water flow, and the accumulation of deposit on the nail spacer might promote local overheating at the nail spacer and increase the local tube temperature that resulted in instant bulging and thin-lip rupture of the tube. The cap leakage might be prevented by ensuring the cap's dimensions fulfilled the design requirement and using low alloy carbon steel containing chromium such as ASTM A213 Grade T12, the same material as the un-leak cap.

## Declarations

### Author contribution statement

Husaini Ardy: Conceived and designed the experiments; Analyzed and interpreted the data; Wrote the paper.

Yudhistira Perdana Putra: Performed the experiments; Analyzed and interpreted the data; Contributed reagents, materials, analysis tools or data.

Adimas Dwi Anggoro & Arie Wibowo: Analyzed and interpreted the data; Wrote the paper.

### Funding statement

Yudhistira Perdana Putra was supported by PT. Pupuk Kaltim, Bontang Indonesia.

### Data availability statement

Data included in article/supp. material/referenced in article.

### Declaration of interests statement

The authors declare no conflict of interest.

### Additional information

No additional information is available for this paper.
